# Supplementary material for: Evaluation of physical activity calorie equivalent (PACE) labels’ impact on energy purchased in cafeterias: A stepped-wedge randomised controlled trial
Source: PLoS Med. 2022 Nov 8;19(11):e1004116. doi: 10.1371/journal.pmed.1004116 (PMC9642872; doi:10.1371/journal.pmed.1004116)
Supplement: S1 Study Protocol — (DOCX) [file pmed.1004116.s002.docx]

STUDY PROTOCOL

Effect of physical activity calorie equivalent (PACE) labels on energy purchased in cafeterias: protocol for a stepped-wedge randomised controlled trial

James P Reynolds*, Minna Ventsel, Alice Hobson, Mark Pilling, Rachel Pechey, Susan Jebb, Gareth J Hollands, Theresa M Marteau*

# Abstract

## Background

Overconsumption of energy from food contributes to high rates of overweight and obesity in many populations. A recent systematic review suggested that PACE labels may be effective at reducing energy selected and consumed, yet few of the studies were conducted in real-world settings.

## Methods

A stepped-wedge randomised controlled trial to assess the impact of PACE labels on energy purchased from food and non-alcoholic drinks. The trial will take place in 10 cafeterias. The primary outcome is the total energy (kcal) purchased per day from intervention items using a pooled effect across all sites.

## Discussion

This study will provide the most robust estimate to date of the effect size of PACE labels on the energy of food and drink purchased in cafeterias.

## Study registration

ISRCTN and OSF

*Authors for correspondence: James P Reynolds, jpr63@medschl.cam.ac.uk;Theresa M Marteau, tm388@cam.ac.uk

# Background

Unhealthy patterns of food consumption including excess energy intake have contributed to over 60% of the UK adult population being overweight or obese, contributing to high and rising levels of type 2 diabetes and 13 different types of cancer [1,2]. One approach to reducing excess energy intake has been to add labels on food and drinks to inform people about the energy composition of the product. A meta-analysis of three calorie labelling studies in restaurants suggests a reduction in energy purchased by 47kcals per meal [3]. However, the quantity and quality of this evidence is limited. Two recent randomised trials in worksite cafeterias, which are more relevant to the current study, suggest that calorie labelling has no discernible effect on energy purchased [4,5].

An alternative to providing calorie labels is to convert calorie information into the physical activity needed to expend the energy in that product. These labels, known as PACE (Physical Activity Calorie Equivalent) labels, typically include an image to highlight the type of physical activity – usually walking or running – together with information on how much of this activity is required. A recent systematic review concluded that PACE labels may reduce energy selected from menus and decrease the energy consumed when compared to no labelling or other types of labelling such as calorie labelling [6]. However, of the 15 included studies, most were of an unclear risk of bias and only one was conducted in a real-world setting [7]. The remaining 14 studies were conducted online (n = 8) or in laboratory settings (n = 6). The one field study only examined the effect of PACE labels on the purchase of sugar-sweetened beverages. Further field studies are therefore needed to estimate the effectiveness of PACE labels on purchasing and consumption of food and non-alcoholic drinks.

PACE labels take many forms (see Appendix A) yet there is an absence of evidence regarding their relative effectiveness. Online studies have investigated the effects of framing the physical activity equivalent in terms of miles vs minutes, the effects of adding an image depicting the activity, and the effects of including kcals along with the PACE information. However, there is currently no evidence that any of these variants strengthen intentions to change behaviour [8–11].

In the absence of evidence to inform the characteristics of the PACE label to be used in the current study, the investigators judged the most accessible and easily understood design, namely communicating the number of minutes the average person would need to spend walking to expend the calories contained in the product. This will be presented with an image of a figure walking, as used in previous research, along with the energy content of the product expressed in kcals.

# Study aim and hypotheses

*Aim:* To estimate the impact on daily energy purchased of PACE labels in cafeterias.

*Hypothesis 1:* PACE labels will reduce energy purchased relative to no labels

# Methods

## Cafeterias

We aim to recruit ten cafeterias based in England to participate. Cafeterias will be approached opportunistically and assessed for eligibility based on criteria set for similar earlier studies [12]:

1. The cafeteria must cater to a workforce greater than 500
2. having electronic point of sale (EPOS) tills that record sales data
3. being able to provide kcal information for all food and drink being sold
4. an absence of existing calorie labels (not including nutritional information on branded packaging)

The PACE label systematic review [6] did not include any studies assessing the impact of PACE labels on purchasing or consumption of food or non-alcoholic drinks in real world settings. It is therefore not possible to estimate from this review the required number of sites needed to detect a reported effect. We aim to recruit 10 sites, which would be more than similar calorie labelling studies in cafeterias [4,5]. This would provide 80% power to detect a change in energy purchase of Cohen’s *d* = 1.00, using a before-after repeated measures design with 4-weeks in each period, using a 2-sided test and at the 5% significance level using a paired t-test.

## Study design

A stepped-wedge design [13] will be used, with each of the sites randomly allocated to the time at which they implement the PACE label intervention**.**

Weeks one to four will comprise the minimum baseline period during which time data will be recorded without any intervention. This will be followed by a period of 8 weeks during which the interventions will be introduced – in an order determined by randomisation - and maintained until the end of the trial period.

The PACE label intervention will be introduced in week five at the first two sites. After this, two sites per week will introduce the intervention. The interventions will be maintained until the end of week 12 when data collection ends across all sites. This means baseline and intervention periods last between 4 and 8 weeks (see Appendix B).

## Randomisation

Randomisation will be performed by a statistician (MP) allocating a list of anonymised site names using random numbers.

# Measures

## Primary outcome

Total energy (kcal) purchased from intervention food categories per day after controlling for the total transactions. Total energy purchased is calculated from the total number of sales for all items within the intervention categories and the total number of calories for each of these items. Sales data are recorded using electronic tills every day of operation during the trial.

Transactions are defined as the number of unique payments to purchase products in the cafeteria, whereas sales are defined as the total number of individual products that are sold in the cafeteria.

## Secondary outcomes

1. Total energy (kcal) purchased per day from i. non-intervention food and drink categories, and ii. all food and drink products. Total energy purchased is calculated from the total number of sales and the total number of calories for each of these items.
2. Total revenue from each cafeteria. This is calculated from the number of all items sold in the cafeterias and the price of each of these items.

## Additional measures

Demographic characteristics of the employees based at each site will be requested from the employer(s), including age, gender, and occupational status.

Further variables may include the day of the week and notable events (e.g., a workplace party) that may affect sales.

Information specific to the intervention: mean energy (kcals) per item during baseline, mean energy (kcals) per item during the PACE label intervention.

# Physical activity calorie equivalent (PACE) labels

The intervention is an Information x Product intervention in the TIPPME typology [14].

PACE labels contain i. information on the energy content (kcal) of a product, and ii. information on the amount of physical activity required to expend this amount of energy (see Appendix C for example). The physical activity equivalent in the current study will be expressed in terms of minutes of walking required to expend the energy (kcals) contained within the product. An image of a figure walking and the energy (kcal) content of the product will also be displayed with this information. The formula used to calculate the PACE values is described in Appendix D.

The labels will be displayed in up to four places:

1. Attached on the product itself where this is appropriate (e.g., a muffin made on site wrapped in plastic). This will not be possible in certain cases where there is not a place to attach it or the product is branded (e.g., a hot meal; a can of coke; a packet of crisps).
2. Along shelf edging at the point of choice
3. On tent cards placed next to products
4. On menus (printed or electronic via email or screens)

## Intervention categories for the PACE label intervention

Intervention categories are defined as the target food and drink categories and products within those that receive the labels. This will depend on discussions with cafeteria managers and catering companies but will likely include the following:

*Main meals*: the meat or vegetarian principal element of a meal

*Sides*: carbohydrate-rich portions (*e.g.,* chips)

*Sandwiches*: sandwiches, paninis, wraps, bread rolls

*Desserts:* hot desserts (*e.g.,* crumbles), dessert pots (*e.g.,* yoghurt, cheesecake, mousse, jelly, granola) and sliced cake

*Bakery*: freshly made cakes, muffins, cookies. Pre-packed croissants and flapjacks

*Savoury snacks:* crisps

*Confectionery*: chocolate bars, sweets

*Cold drinks*: soft drinks (*e.g.,* can of coke), bottled water

Items that are unlikely to receive the interventions are salads, hot drinks, and items in vending machines.

## Fidelity checks

Regular checks will be performed to ensure each cafeteria has implemented the intervention as planned. The original plan was for members of the research team to visit each site at regular intervals, however due to the COVID-19 pandemic the checks will now be conducted digitally. This involves a staff member at each cafeteria taking photos of the food, drinks, and labels every week and sending them to the research team to conduct checks. If concerns are raised about the implementation of the intervention during this visit the following occurs:

i. The researcher sends a detailed description of her/his concerns to the study manager (JR);

ii. The site representative is contacted with a view to addressing the concern that working day and with instruction to send photos confirming the change has been made;

iii. If no evidence of a change has been provided, the catering company’s senior manager is contacted and requested to intervene.

These checks along with regular communications with a manager at each site are also conducted to promote site retention and engagement.

Adherence to or violation of the intervention will be recorded for potential use in secondary analyses.

## Data analysis / statistical plan

Generalised linear mixed models (or similar, depending on model checks) will be used to estimate the potential impact of the PACE label intervention compared to baseline. The primary analysis will determine the effectiveness of the intervention across all sites, with follow up tests to determine if the effect varied across sites. Primary analyses will be on an intention to treat basis (i.e. data will be analysed according to the period that they should be in [baseline or PACE], regardless of adherence to the intervention).

# Research governance

## Ethical considerations and informed consent

Ethics approval will be sought from the Psychology Research Ethics Committee based at the University of Cambridge.

## Insurance

The University of Cambridge arranges insurance cover for legal liability to pay damages for injury to volunteers participating in the study which has been caused by the University or its employees.

## Safety

We do not anticipate any risks to participants. However, cafeteria representatives will be given a contact email address to raise any concerns or ask questions to researchers.

## Incident reporting

Incident reporting will follow the University of Cambridge procedures. Incidents will be documented and followed up until resolved if possible. At the end of the study a safety report will be compiled and sent to the Principal Investigator (PI) listing all incidents. The Cambridge Psychology Research Ethics Committee will be notified of breaches as appropriate.

## Data management

All aspects of the General Data Protection Regulation, Data Protection Act 2018 and the Freedom of Information Act 2000 will be adhered to. No personal data will be collected.

### Participant Identifiable Data (PID)

Aggregated sales data will be collected from each site. The researchers will not have access to individual level data. Data that identifies the cafeterias will be anonymised after data analysis has been completed.

### Anonymous study data

Electronic data will be anonymised by a unique study identifier. Anonymous study data will be held on network drives. Computer data files will be regularly backed up on a University of Cambridge network drive.

### Data sharing

Anonymous study data may be shared with collaborators for the purposes of analysis and results interpretation under appropriate collaboration agreements.

### Long term data archiving

At the end of the study, electronic study data (including finalised anonymous data sheet) will be transferred to a designated storage facility for long-term archiving. Study data will be kept for a minimum of 20 years.

### Open data

The data will not made freely available as this is proprietary data provided in confidence from a business.

### Participant drop out

Aggregated sales data will be collected from each site and therefore we will not have access to individual level data.

### Revoked data

Aggregated sales data will be collected from each site and therefore we will not have access to individual level data. Therefore, individual data cannot be revoked.

## Quality control and quality assurance

The study team will be responsible for data quality.

# Publication policy

The findings from this research study will be published in an appropriate scientific journal, made available open access, and/or presented at an appropriate meeting. Study data will be collected and held by the study investigators. The datasets generated during and/or analysed during the current study are not expected to be made available as they are commercially sensitive, and are to be provided by the participating cafeterias on condition that they are not shared beyond the research team.

# Study personnel

James Reynolds

Research Associate

Behaviour and Health Research Unit

University of Cambridge

East Forvie Site

Cambridge CB2 0SR

Tel: +44 (0)1223 762569

Email: [jpr63@medschl.cam.ac.uk](mailto:jpr63@medschl.cam.ac.uk)

Minna Ventsel

Research Assistant

Behaviour and Health Research Unit

University of Cambridge

East Forvie Site

Cambridge CB2 0SR

Tel: +44 (0)1223 762510

Email: [mv491@medschl.cam.ac.uk](mailto:mv491@medschl.cam.ac.uk)

Alice Hobson

Research Assistant

Behaviour and Health Research Unit

University of Cambridge

East Forvie Site

Cambridge CB2 0SR

Tel: +44 (0)1223 762510

Email: [aeh86@medschl.cam.ac.uk](mailto:aeh86@medschl.cam.ac.uk)

Mark Pilling

Senior Research Associate in Statistics

Behaviour and Health Research Unit

University of Cambridge

East Forvie Site

Cambridge CB2 0SR

Email: [mark.pilling@medschl.cam.ac.uk](mailto:mark.pilling@medschl.cam.ac.uk)

Susan Jebb

Professor

Nuffield Department of Primary Care Health Sciences

University of Oxford

Radcliffe Primary Care Building

Woodstock Road

Oxford, OX2 6GG

Tel: +44 (0)1865 617826

Email: [susan.jebb@phc.ox.ac.uk](mailto:susan.jebb@phc.ox.ac.uk)

Rachel Pechey

Epidemiologist

Nuffield Department of Primary Care Health Sciences

University of Oxford

Radcliffe Primary Care Building

Woodstock Road

Oxford, OX2 6GG

Email: [rachel.pechey@phc.ox.ac.uk](mailto:rachel.pechey@phc.ox.ac.uk)

Gareth Hollands

Senior Research Associate

Behaviour and Health Research Unit

University of Cambridge

East Forvie Site

Cambridge CB2 0SR

Tel: +44 (0)1223 (7)30318

Email: [gjh44@medschl.cam.ac.uk](mailto:gjh44@medschl.cam.ac.uk)

Theresa Marteau

Director

Behaviour and Health Research Unit

University of Cambridge

East Forvie Site

Cambridge CB2 0SR

Tel: +44 (0)1223330562

Email: [tm388@medschl.cam.ac.uk](mailto:tm388@medschl.cam.ac.uk)

# Funding source

Collaborative Award in Science from Wellcome Trust (Behaviour Change by Design: 206853/Z/17/Z) awarded to Theresa Marteau, Paul Fletcher, Gareth Hollands and Marcus Munafò. The funder will not be involved in the study design or data analysis.

# Conflicts of Interest

The study investigators have no known conflicts of interest to declare.

# Appendices

**Appendix A.** Characteristics of PACE labels from studies reported in Daley et al (2019)

| Author (year) | Form of exercise | Unit of energy (miles/minutes) | Includes calories | Includes image |
| --- | --- | --- | --- | --- |
| Antonelli & Viera (2015) | walking | miles and minutes (separate conditions) | yes | yes |
| Downs, Wisdom & Loewenstein (2015) | Study 1. running on a treadmill | Study 1. minutes | Unclear | Unclear |
| Dowray et al. (2013) | walking | miles and minutes (separate conditions) | yes | yes |
| Lee & Thompson (2016) | walking | miles | yes | yes |
| Masic, Christiansen & Boyland (2017) | walking | minutes | condition 1 (no), condition 2 (yes) | no |
| Shah et al. (2016) | brisk walking | minutes | Unclear | Unclear |
| Pang & Hammond (2013) | running | minutes | yes | no |
| Reale & Flint (2016) | walking | minutes | Unclear | Unclear |
| Hartley, Keast & Liem (2018) | walking | minutes | no | yes |
| Platkin et al. (2014) | walking | minutes | yes | no |
| James, Adams-Huet & Shah (2015) | brisk walking | minutes | Unclear | Unclear |
| Montford, Peloza & Goldsmith (2017) | Study 1. rowing  Study 2. spinning  Study 3. condition 1 (walking), condition 2 (spinning)  Study 4. running | minutes | Study 1. condition 1 (yes), condition 2 (Unclear)  Study 2. Unclear  Study 3. Unclear  Study 4. Unclear | Study 1. condition 1 (no), condition 2 (yes)  Study 2. yes  Study 3. Unclear  Study 4. Unclear |
| Bleich et al. (2012) | jogging | minutes | no | Unclear |
| Hartley, Keast & Liem (2019) | walking | minutes | no | yes |

**Appendix B.** Study design


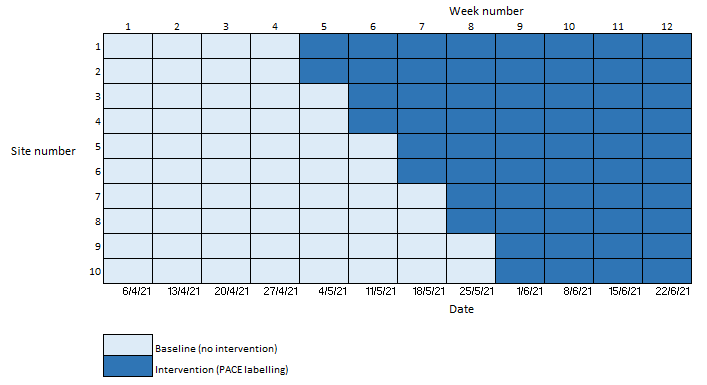


**Appendix** **C.** Example of PACE labelling


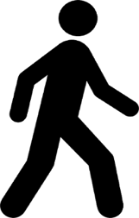


35 minutes

walking

Walkers salt & vinegar crisps

129 CALORIES

£0.80

=

**Appendix** **D.** PACE formula

Kcal of product / 3.65 = minutes required to expend energy

**Example:**

Crisps = 129 kcals

Minutes to expend energy = 150 / 3.65 = 35 minutes of walking

**Formula breakdown:**

**Weight**: We used the mean weight of men (85.4kg) and women (72.1kg) based on data from 2019 [15]

**Age**: 40.2. Median age from England and Wales from 2019 [16]

**BMR (Basal metabolic rate):** 1641.5

Source: <http://www.fao.org/3/y5686e/y5686e07.htm>

Female:

8.126 * kg + 845.6 = (8.126* 72.1) + 845.6 = 1431

Male:

11.472 * kg + 873.1 = (11.472 * 85.4) + 873.1 = 1852

Average: (1852+1431)/2 = 1641.5

**PAR (Physical activity ratio for walking):** 3.2

Source: <http://www.fao.org/3/y5686e/y5686e07.htm>

**TEE (total energy expenditure)** = PAR * BAR = 3.2 * 1641.5 = 5253 kcal/day

**TEE** = 5253/(24 hours * 60 minutes) = 3.65 kcals/minute

# Reference

##

1. Steel N, Ford JA, Newton JN, Davis AC, Vos T, Naghavi M, et al. Changes in health in the countries of the UK and 150 English Local Authority areas 1990–2016: a systematic analysis for the Global Burden of Disease Study 2016. The Lancet. 2018;392:1647–61.

2. Swinburn BA, Kraak VI, Allender S, Atkins VJ, Baker PI, Bogard JR, et al. The global syndemic of obesity, undernutrition, and climate change: The Lancet Commission report. The Lancet. 2019;393(10173):791–846.

3. Crockett RA, King SE, Marteau TM, Prevost AT, Bignardi G, Roberts NW, et al. Nutritional labelling for healthier food or non‐alcoholic drink purchasing and consumption. Cochrane Database Syst Rev. 2018;(2).

4. Vasiljevic M, Cartwright E, Pilling M, Lee M, Bignardi G, Pechey R, et al. Impact of calorie labelling in worksite cafeterias: a stepped wedge randomised controlled pilot trial. Int J Behav Nutr Phys Act. 2018;15(1):41.

5. Vasiljevic M, Fuller G, Pilling M, Hollands GJ, Pechey R, Jebb SA, et al. What is the impact of increasing the prominence of calorie labelling? A stepped wedge randomised controlled pilot trial in worksite cafeterias. Appetite. 2019;141(1):104304.

6. Daley AJ, McGee E, Bayliss S, Coombe A, Parretti HM. Effects of physical activity calorie equivalent food labelling to reduce food selection and consumption: systematic review and meta-analysis of randomised controlled studies. J Epidemiol Community Health. 2019;74(3):269–75.

7. Bleich SN, Herring BJ, Flagg DD, Gary-Webb TL. Reduction in Purchases of Sugar-Sweetened Beverages Among Low-Income Black Adolescents After Exposure to Caloric Information. Am J Public Health. 2011 Dec 15;102(2):329–35.

8. Antonelli R, Viera AJ. Potential Effect of Physical Activity Calorie Equivalent (PACE) Labeling on Adult Fast Food Ordering and Exercise. PLoS ONE [Internet]. 2015 Jul 29 [cited 2020 Feb 20];10(7). Available from: https://www.ncbi.nlm.nih.gov/pmc/articles/PMC4519110/

9. Dowray S, Swartz JJ, Braxton D, Viera AJ. Potential effect of physical activity based menu labels on the calorie content of selected fast food meals. Appetite. 2013 Mar 1;62:173–81.

10. Masic U, Christiansen P, Boyland EJ. The influence of calorie and physical activity labelling on snack and beverage choices. Appetite. 2017 May 1;112:52–8.

11. Montford WJ, Peloza J, Goldsmith RE. No pain, no gain: how PACE information attenuates consumption. J Consum Mark. 2017 Jan 1;34(7):525–40.

12. Reynolds JP, Ventsel M, Kosīte D, Rigby Dames B, Brocklebank L, Masterton S, et al. Impact of decreasing the proportion of higher energy foods and reducing portion sizes on food purchased in worksite cafeterias: A stepped-wedge randomised controlled trial. PLoS Med. 2021 Sep 14;18(9):e1003743.

13. Campbell MJ, Walters SJ. How to design, analyse and report cluster randomised trials in medicine and health related research. John Wiley & Sons; 2014.

14. Hollands GJ, Bignardi G, Johnston M, Kelly MP, Ogilvie D, Petticrew M, et al. The TIPPME intervention typology for changing environments to change behaviour. Nat Hum Behav. 2017;1(8):0140.

15. NHS Digital. Health Survey for England 2019 [Internet]. 2020 [cited 2021 Feb 19]. Available from: https://digital.nhs.uk/data-and-information/publications/statistical/health-survey-for-england/2019

16. ONS. Population estimates by output areas, electoral, health and other geographies, England and Wales - Office for National Statistics [Internet]. 2020 [cited 2021 Feb 22]. Available from: https://www.ons.gov.uk/peoplepopulationandcommunity/populationandmigration/populationestimates/bulletins/annualsmallareapopulationestimates/mid2019
