# Supplementary material for: Evaluation of physical activity calorie equivalent (PACE) labels’ impact on energy purchased in cafeterias: A stepped-wedge randomised controlled trial
Source: PLoS Med. 2022 Nov 8;19(11):e1004116. doi: 10.1371/journal.pmed.1004116 (PMC9642872; doi:10.1371/journal.pmed.1004116)
Supplement: S1 Additional Data — (DOCX) [file pmed.1004116.s003.docx]

| Site | Hot food | Sandwiches | Breakfast | Bakery | Confectionery | Savoury Snacks | Hot drinks | Cold drinks | Fruit and Veg | Salads/ Cold Snacks | Ice Cream | Grocery | Condiments | Effect of intervention |
| --- | --- | --- | --- | --- | --- | --- | --- | --- | --- | --- | --- | --- | --- | --- |
| 1 | 2 | 19 | 6 | 11 | 15 | 26 | 13 | 6 | 0 | 0 | 1 | 1 | 1 | -39% |
| 9a | 10 | 5 | 22 | 8 | 8 | 15 | 4 | 5 | 20 | 0 | 0 | 0 | 2 | -29% |
| 2 | 15 | 5 | 15 | 7 | 8 | 13 | 5 | 6 | 21 | 1 | 1 | 0 | 4 | -24% |
| 10 | 10 | 14 | 23 | 6 | 7 | 15 | 10 | 5 | 7 | 0 | 0 | 0 | 1 | -15% |
| 3 | 8 | 6 | 29 | 5 | 5 | 10 | 8 | 7 | 9 | 1 | 0 | 0 | 11 | 1% |
| 6 | 6 | 6 | 58 | 2 | 3 | 7 | 3 | 3 | 8 | 0 | 0 | 0 | 3 | 5% |
| 7a | 22 | 4 | 11 | 9 | 6 | 8 | 4 | 5 | 27 | 1 | 0 | 0 | 3 | 5% |
| 8 | 13 | 6 | 34 | 11 | 4 | 3 | 2 | 8 | 14 | 0 | 0 | 0 | 4 | 7% |
| 5a | 27 | 5 | 12 | 4 | 3 | 9 | 2 | 4 | 29 | 0 | 1 | 0 | 3 | 7% |
| 4 | 27 | 7 | 5 | 12 | 8 | 9 | 0 | 7 | 22 | 2 | 0 | 0 | 1 | 17% |

Table A. Proportion (%) of kcals purchased from each food category by site [Ordered by effect size]

Table B. Exploratory correlations between the effect of PACE on energy purchased from intervention items at each site and the proportion (%) of kcals purchased from each food category at each site (n = 10)

|  | Hot food | | Sandwiches | | Breakfast | | Bakery | | Confectionery | | Savoury Snacks | | Hot drinks | | Cold drinks | | Fruit and Veg | |
| --- | --- | --- | --- | --- | --- | --- | --- | --- | --- | --- | --- | --- | --- | --- | --- | --- | --- | --- |
|  | *r* | *p* | *r* | *p* | *r* | *p* | *r* | *p* | *r* | *p* | *r* | *p* | *r* | *p* | *r* | *p* | *r* | *p* |
| Effect of interventions | .63 | .053 | -.56 | .089 | .22 | .547 | -.11 | .759 | -.74 | .014 | -.86 | .001 | -.72 | .019 | .06 | .863 | .43 | .763 |

*Note.* Negative correlations imply that the PACE interventions where more effective in cafeterias where they sold a lot of the products from the associated category.

Table C. Fidelity to protocol [Ordered by effect size]

| Cafeteria | Proportion (%) of total products that received PACE labels | Effect of intervention |
| --- | --- | --- |
| 1 | 97 | -39% |
| 9 | 84 | -29% |
| 2 | 100 | -24% |
| 10 | 90 | -15% |
| 3 | 92 | 1% |
| 6 | 96 | 5% |
| 7 | 90 | 5% |
| 8 | 92 | 7% |
| 5 | 95 | 7% |
| 4 | 93 | 17% |

| Cafeterias | Number of employees (mean) | Proportion male (%) | Mean age | Job roles | Proportion working full time (%) | Effect of intervention |
| --- | --- | --- | --- | --- | --- | --- |
| 1 | 1800 | 70 | 37 | Pilots/Office/cleaners/training/Admin/facilities/Managers | 80 | -39% |
| 9 | 750 | 66 | 42 | Drivers/Office/cleaners/Sales/Admin/Delivery/Managers | 40 | -29% |
| 2 | 697 | 75 | 47 | Processing/ Drivers Office Staff | 62 | -24% |
| 10 | 7200 | - | - | - | - | -15% |
| 3 | 1500 | 80 | 44 | Engineers/designers/accountants/facilities/management | 100 | 1% |
| 6 | 3473 | 95 | 39 | Manufacturing associates/Admin & Management | 97 | 5% |
| 7 | 500 | 60 | 47 | Processing/ Drivers Office Staff | - | 5% |
| 8 | 230 | 50 | 30 | Manufacturing/ Management & Office | 100 | 7% |
| 5 | 850 | 70 | 45 | Processing/ Drivers Office Staff | 60 | 7% |
| 4 | 2361 | 80 | 26 | - | - | 17% |

Table D. Demographic characteristics of employees at each site [Ordered by effect size]

Figure E. Kcals purchased from intervention items per day of trial, with separate trend lines before (black) and after (blue) the PACE intervention was implemented


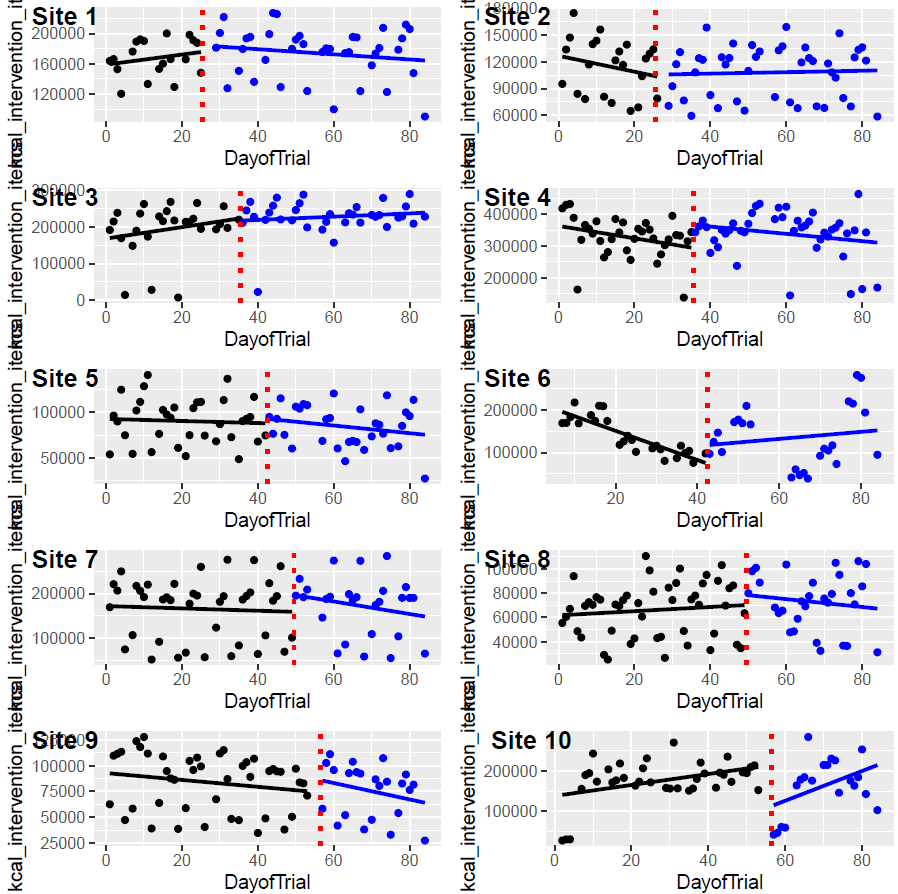

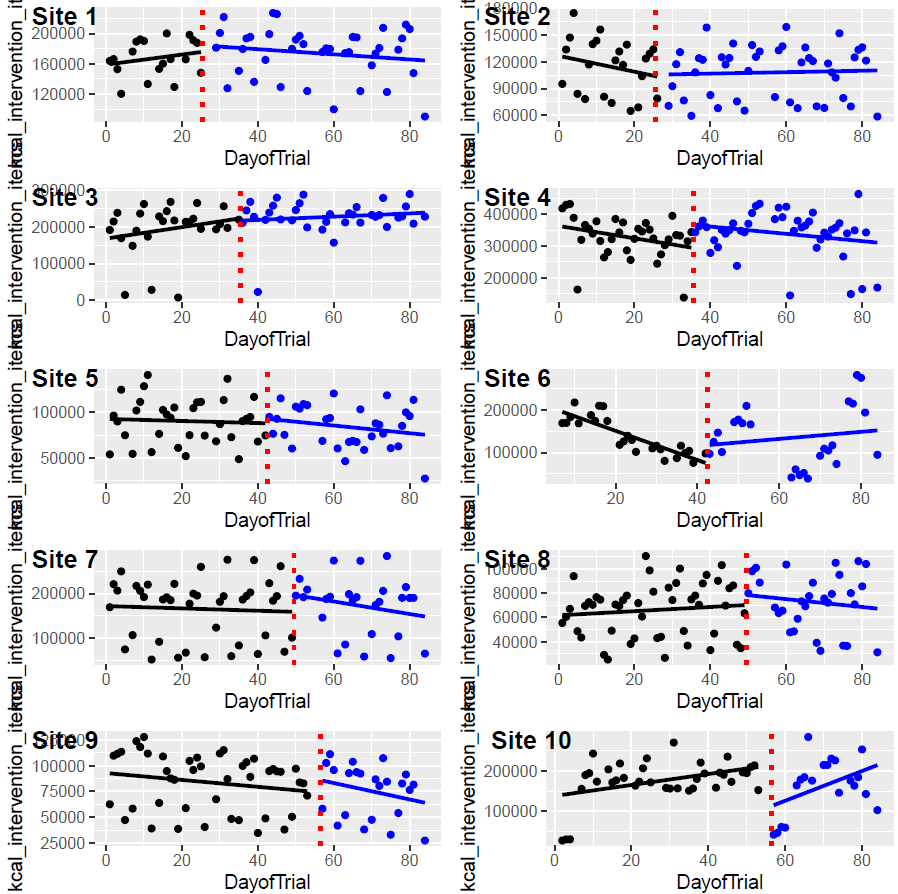


Table F. Sensitivity analysis 1 full model. Analysis in which we remove some data from the cafeteria that did not provide timely evidence of intervention implementation

| Overall model | | |  |  |
| --- | --- | --- | --- | --- |
| Modelling of the mean (identity link) | | |  |  |
|  | |  |  |  |
|  | Estimate | Std. Error | t value | Pr(>\|t\|) |
| (Intercept) | 23508 | 1568.14 | 14.99 | < .001 |
| PACE | -1939.69 | 1287.57 | -1.51 | 0.132 |
| Transactions | 412.62 | 6.65 | 62.06 | < .001 |
| Day from start on study period | -113.09 | 54.53 | -2.07 | 0.0385 |
| Modelling of the variance (log link) | | |  |  |
|  | Estimate | Std. Error | t value | Pr(>\|t\|) |
| (Intercept) | 11.07 | 0.1015 | 109.06 | < .001 |
| Site 2 (Ref=Site 1) | -0.6597 | 0.1272 | -5.186 | < .001 |
| Site 3 | -1.1434 | 0.1412 | -8.096 | < .001 |
| Site 4 | -0.0997 | 0.1516 | -0.658 | 0.223 |
| Site 5 | -1.3881 | 0.1384 | -10.030 | < .001 |
| Site 6 | -1.2162 | 0.1622 | -7.513 | < .001 |
| Site 7 | -0.8174 | 0.1417 | -5.770 | < .001 |
| Site 8 | -1.8415 | 0.1355 | -13.589 | < .001 |
| Site 9 | -0.8961 | 0.1297 | -6.910 | < .001 |
| Site 10 | -0.9194 | 0.1323 | -6.948 | < .001 |
|  |  |  |  |  |
| By Site: |  |  |  |  |
|  |  |  |  |  |
| ------------------------------------------------------------------ | | | | |
| Modelling of the mean (identity link) | | |  |  |
| Mu Coefficients: | |  |  |  |
|  | Estimate | Std. Error | t value | Pr(>\|t\|) |
| (Intercept) | 20996.59 | 2127.91 | 9.867 | < .001 |
| Site 1 | -65204.33 | 6381.121 | -10.218 | < .001 |
| Site 2 | -27964.97 | 3836.01 | -7.290 | < .001 |
| Site 3 | 1663.83 | 4498.22 | 0.370 | 0.712 |
| Site 4 | 54491.71 | 7567.12 | 7.201 | < .001 |
| Site 5 | 6543.10 | 3057.03 | 2.140 | 0.033 |
| Site 6 | 6571.41 | 4176.44 | 1.573 | 0.116 |
| Site 7 | 7739.99 | 5850.38 | 1.323 | 0.186 |
| Site 8 | 4813.25 | 2247.66 | 2.141 | 0.033 |
| Site 9 | -24694.52 | 4454.17 | -5.544 | < .001 |
| Site 10 | -27078.60 | 4968.54 | -5.450 | < .001 |
| Transactions | 412.94 | 7.71 | 53.537 | < .001 |
| Day from start on study period | -102.07 | 58.03 | -1.759 | 0.079 |
| Modelling of the variance (log link) | | |  |  |
|  | Estimate | Std. Error | t value | Pr(>\|t\|) |
| (Intercept) | 10.447 | 0.1097 | 95.262 | < .001 |
| Site 2 (Ref=Site 1) | -0.3944 | 0.1293 | -3.050 | 0.002 |
| Site 3 | -0.4999 | 0.1499 | -3.336 | < .001 |
| Site 4 | 0.2132 | 0.1586 | 1.344 | 0.179 |
| Site 5 | -0.7686 | 0.1455 | -5.281 | < .001 |
| Site 6 | -0.5824 | 0.1726 | -3.374 | < .001 |
| Site 7 | -0.1909 | 0.1510 | -1.265 | 0.207 |
| Site 8 | -1.2327 | 0.1431 | -8.617 | < .001 |
| Site 9 | -0.5326 | 0.1338 | -3.980 | < .001 |
| Site 10 | -0.5827 | 0.1366 | -4.265 | 0.001 |

Table G. Sensitivity analysis 2 full model. For the analysis in which the energy estimates were not adjusted for incorrect button presses.

|  | | |  |  |
| --- | --- | --- | --- | --- |
| Overall model | | |  |  |
| Modelling of the mean (identity link) | |  |  |  |
|  | Estimate | Std. Error | t value | Pr(>\|t\|) |
| (Intercept) | 23508.5 | 1648.88 | 14.257 | <2e-16 |
| PACE | -1939.69 | 1701.43 | -1.14 | 0.2547 |
| Transactions | 412.621 | 7.306 | 56.479 | <2e-16 |
| Day from start on study period | -113.092 | 57.203 | -1.977 | 0.0485 |
| --- |  |  |  |  |
| Modelling of the variance (log link) | | |  |  |
|  | Estimate | Std. Error | t value | Pr(>\|t\|) |
| (Intercept) | 11.07148 | 0.10165 | 108.92 | < 2e-16 |
| Site 2 (Ref=Site 1) | -0.65969 | 0.1278 | -5.162 | 3.26E-07 |
| Site 3 | -1.14337 | 0.14134 | -8.09 | 3E-15 |
| Site 4 | -0.09971 | 0.15252 | -0.654 | 0.514 |
| Site 5 | -1.3881 | 0.13837 | -10.032 | < 2e-16 |
| Site 6 | -1.21615 | 0.16251 | -7.484 | 2.39E-13 |
| Site 7 | -0.81744 | 0.14223 | -5.747 | 1.4E-08 |
| Site 8 | -1.8415 | 0.13526 | -13.615 | < 2e-16 |
| Site 9 | -0.89609 | 0.12988 | -6.9 | 1.26E-11 |
| Site 10 | -0.91938 | 0.13233 | -6.947 | 9.16E-12 |
| --- |  |  |  |  |
| By Site: |  |  |  |  |
| Modelling of the mean (identity link) | | |  |  |
|  | Estimate | Std. Error | t value | Pr(>\|t\|) |
| (Intercept) | 20996.59 | 2127.82 | 9.868 | < 2e-16 |
| Site 1 | -65204.3 | 6355.16 | -10.26 | < 2e-16 |
| Site 2 | -27965 | 3825.81 | -7.31 | 8.13E-13 |
| Site 3 | 1663.826 | 4439.279 | 0.375 | 0.7079 |
| Site 4 | 54491.71 | 7532.228 | 7.234 | 1.36E-12 |
| Site 5 | 6543.104 | 3053.642 | 2.143 | 0.0325 |
| Site 6 | 6571.414 | 4163.452 | 1.578 | 0.115 |
| Site 7 | 7739.994 | 5833.889 | 1.327 | 0.1851 |
| Site 8 | 4813.245 | 2245.838 | 2.143 | 0.0325 |
| Site 9 | -24694.5 | 4449.226 | -5.55 | 4.2E-08 |
| Site 10 | -27078.6 | 4945.763 | -5.475 | 6.31E-08 |
| Transactions | 412.943 | 7.615 | 54.224 | < 2e-16 |
| Day from start on study period | -102.072 | 57.985 | -1.76 | 0.0788 |
| --- |  |  |  |  |
| Modelling of the variance (log link) | | |  |  |
|  | |  |  |  |
|  | Estimate | Std. Error | t value | Pr(>\|t\|) |
| (Intercept) | 10.4468 | 0.1074 | 97.274 | < 2e-16 |
| Site 2 (Ref=Site 1) | -0.3944 | 0.1283 | -3.073 | 0.002207 |
| Site 3 | -0.4999 | 0.1477 | -3.385 | 0.000757 |
| Site 4 | 0.2132 | 0.1556 | 1.37 | 0.171071 |
| Site 5 | -0.7686 | 0.1434 | -5.36 | 1.17E-07 |
| Site 6 | -0.5824 | 0.1684 | -3.458 | 0.000581 |
| Site 7 | -0.1909 | 0.1484 | -1.287 | 0.198738 |
| Site 8 | -1.2327 | 0.1409 | -8.748 | < 2e-16 |
| Site 9 | -0.5326 | 0.1329 | -4.006 | 6.9E-05 |
| Site 10 | -0.5827 | 0.1355 | -4.3 | 1.98E-05 |
