# Supplementary material for: Evaluation of physical activity calorie equivalent (PACE) labels’ impact on energy purchased in cafeterias: A stepped-wedge randomised controlled trial
Source: PLoS Med. 2022 Nov 8;19(11):e1004116. doi: 10.1371/journal.pmed.1004116 (PMC9642872; doi:10.1371/journal.pmed.1004116)
Supplement: Attachment — Submitted filename: 220609 Responses to reviewers.docx [file pmed.1004116.s004.docx]

**Requests from the editors:

Please adapt the title to begin "Evaluation of ...", crafting the other wording as needed.**

*Response:* We have altered the title from:

Effect of physical activity calorie equivalent (PACE) labels on energy purchased in cafeterias: a stepped-wedge randomised controlled trial

To:

**Evaluation** of physical activity calorie equivalent (PACE) labels’ **impact** on energy purchased in cafeterias: a stepped-wedge randomised controlled trial

**Please quote the study dates in your abstract.**

*Response:* We have added this into the abstract:

***“****The study ran for 12 weeks (06 April 21 to 28 June 21)…”* **Also, please add some demographic details (e.g., from table 1) to the abstract.**

*Response:* We have added this into the abstract:

*“**There were approximately 19,000 workers employed at the sites, 72% male, with an average age of 40.”*

**In the abstract and throughout the text, please quote p values alongside 95% CI, where available.**

*Response:* We have added this into the abstract.

**You report some quantitative details and comparisons with findings of other studies in the second paragraph of the Discussion (main text), and it may be that this element should appear at the end of the Results section.**

*Response:* We have reviewed this and we would prefer to keep this in the Discussion, however we have edited the text slightly to remove some repetitive statistics.
 **Throughout the text, please adapt reference call-outs to the following style: "... 13 different types of cancer [1-3]." and restructure the reference list as appropriate.**

*Response:* We have made this change. **Please use the style "... 3 eligibility criteria" throughout, although numbers should be spelt out at the start of sentences.**

*Response:* We have made these changes throughout the whole manuscript.

**In the reference list, please convert all italics into plain text.**

*Response:* We have made this change. **Noting Deery et al, please list no more than 6 author names, followed by "et al.", and remove the ampersands.**

*Response:* We have made this change.
 **Noting Vasiljevic et al, please ensure that all references have full access details.**

*Response:* We have made this change:

*“Vasiljevic M, Fuller G, Pilling M, Hollands GJ, Pechey R, Jebb SA, Marteau TM (2019) What is the impact of increasing the prominence of calorie labelling? A stepped wedge randomised controlled pilot trial in worksite cafeterias. Appetite 141:104304”* **Thank you very much for including the CONSORT checklist, and please adapt this so that individual items are referred to by section and paragraph number (not by line or page numbers as these generally change in the event of publication).**

*Response:* We have updated this.

**Please also include a completed CONSERVE checklist if appropriate.**

*Response:* Thanks for pointing out the CONSERVE checklist as we weren’t familiar with this. It appears that this is appropriate when a trial undergoes large changes due to extenuating circumstances (eg., COVID-19). Although our trial occurred during COVID-19 we also registered the trial during this time and so there were no major changes from our registration. We therefore have not completed this checklist.  **Please include the trial protocol as an attachment, referred to in the Methods section (main text).**

*Response:* We have added this as an attachment. **Comments from the reviewers:

*** Reviewer #1:

This is a large trial answering a novel research question of public health relevance. The manuscript is generally well presented and methodologically strong. I therefore am very supportive of this work and believe a revision would strengthen the manuscript.

Abstract
Inconsistent use of 'ten' vs '10'**

*Response:* Following further advice from the editor we have made this change to “10” except at the start of a sentence, when it is “Ten”. **Introduction
The introduction is concise and summarises existing PACE labelling literature well. However, it would benefit from revising to better set the scene for why the study was important to conduct. For example, the focus here is on the out of home food sector - most kcal label and PACE literature discussed is tested in that setting and the present study was conducted in the out of home food sector. Why should readers of this journal care about the out of home food sector in relation to medicine/public health? The energy content of foods sold in that setting, how often people eat in that setting and recent policy developments which suggest it is now an area of policy interest (e.g. new kcal labelling law in the UK) spring to mind as being relevant. There is plenty of citeable evidence of relevance - a couple of papers addressing this that we have recently published:** [**https://www.bmj.com/content/363/bmj.k4982**](https://www.bmj.com/content/363/bmj.k4982) **and** [**https://www.bmj.com/content/372/bmj.n40**](https://www.bmj.com/content/372/bmj.n40) **as an example, but there may be
other sources the authors prefer to cite. Similarly, I also think the explanation of why PACE has been considered as an alternative (e.g. some theory and also potentially some reference to public calls for PACE labelling vs. it being considered controversial/questioned by some public health groups) would be informative and build a better case for why this is an important study. A relevant source here -** [**https://www.sciencedirect.com/science/article/pii/S0091743521003820**](https://www.sciencedirect.com/science/article/pii/S0091743521003820)

*Response:* Thank you for the recommendations and suggested papers. We have altered the text in three places to highlight these points, including one those suggested papers here along with another two papers that weren’t suggested:

p.4	*“**One key setting in which to intervene is the out-of-home setting including eating establishments such as restaurants, cafes, and schools. This setting is important to target as, in the UK, up to 33% of meals are eaten out-of-home [4] and the energy content of these products is often excessive [5], with one study estimating out-of-home meals to be around 31% more calorie dense than those eaten in the home [6].”*

p.4	“*These labels aim to help consumers understand the energy content of the products and thus reduce excess energy intake [10].”*

p.25-6	*“**However, there are concerns about PACE labels which go beyond their effectiveness. There have been claims that labels containing food energy information could exacerbate existing eating disorders however there is limited evidence to support this claim to our knowledge [23]. Some evidence comes from a randomised controlled trial which suggests that a multi-component intervention which includes PACE labels does not affect emotional eating or uncontrolled eating in a sample where eating disorders were not measured [24]. A pre-post study in university cafeterias that implemented calorie labels found no change in anxiety, body image concerns, or disordered eating among a group at high risk of disordered eating* *[25]. More evidence is needed before firm conclusions can be reached. A further concern is that PACE labels focus on energy content, rather than other indicators of dietary quality, they may be ignored by people who are not attempting to lose or maintain their weight.”*

**Method
There are differences between the protocol and analysis protocol on the OSF vs. what is reported in this manuscript. One example is the eligibility criteria, another example is the planned use of Bayesian analyses. There may be more (I haven't looked at in detail to compare and will leave it to the authors to do so). The authors should include a section somewhere on deviations from the original protocol method and planned analyses.**

*Response:* We have reviewed the protocol and our manuscript and you are correct, there are several inconsistencies in reporting which we have amended. First, eligibility criteria, this was only an inconsistency in terms of reporting (which is now amended), these criteria were applied as described in the protocol:

*“There were 4 eligibility criteria for participation: i. at least 500 employees based at the cafeteria, ii. sales data recorded using electronic point-of-sale tills, iii. able to provide kcal information for all food and drink sold, and iv., an absence of existing calorie labels”* p.6

Second: Bayes analysis. We have now reported the Bayes results in line with the protocol.

*“A Bayes factor was calculated for the primary outcome: Bayes Factor = 1.03, which suggests there was no evidence that the intervention influences energy purchased.”* P.16

Third: Sensitivity analyses. There was a further inconsistency whereby one of our prespecified sensitivity analyses was not conducted, and the reason for this is now reported.

*“There was one main deviation from the pre-specified analysis plan: we did not conduct the second prespecified sensitivity analysis (see Analysis section for details).”* P.5-6

*“This analysis planned to change the definition of “intervention items” from a product containing a label (e.g., crisps) to a product in which any product within its category (e.g., savoury snacks) contained a label. However, as every product category contained at least one product with a label, this would have just replicated the second secondary outcome, Total energy (kcal) purchased from all food and drink products.”* P.15

**Page 8, line 9. 'There were largely no energy labels on shelf edge labels or menus'. Likewise on line 6 'most labels'. This is very vague and given that the study is about how changes to labelling affected consumer behaviour, the authors should provide more detailed information (i.e. numbers) and explain how this information was obtained.**

*Response:* This information was collected using photos sent as part of the fidelity checks. We have reworded this to make it clear that the only energy labelling was part of standardised nutritional labelling, and was not prominent energy content or PACE content:

*“**Photos were checked (see Fidelity section below) to confirm that during the baseline period no PACE labels or other prominent energy labels were in use. During the baseline period, most pre-existing labels and menus only featured the product name and price. There were some standardised front-of-pack nutrition labels on branded products (e.g., Coca-Cola) and in-house products (e.g., muffins) on which the energy content was provided in small print along with other nutritional information. There was no energy information on shelf-edge labels or menus beyond this standardised nutritional information.”* P.8 **More detail on how fidelity checks were done is needed. Did staff take photos of every single food item pre-intervention each week and then each week during the intervention? The article implicitly reads like this at the moment and I could imagine that over the course of the weeks that the intervention was supposed to be present there would be days when the menu changes and staff forget to do this or over time the labels are less likely to still be where they should be. For example, reading table 2 'yes' vs. 'no' answers seem a simplification of likely compliance. If 1 shelf edge label for 1 food product is photographed, is that evidence of a 'Yes' (I'd assume/hope not), or do x% of food items on a shelf need shelf edge labels to get a 'Yes' (presumably so)?**

*Response:* Due to the large number of products on sale that were changed regularly throughout the sites it was not possible to obtain photos of every product on sale each day across the 10 sites. Instead, we requested photos of every product on sale once a week from each site and made assumptions that the photos we received were approximately representative of the broader implementation. The reviewer is correct that the “yes” vs “no” is an oversimplification and that the criteria was 1 photo of the label in that location. In practice, there was only one situation where we only received one photo of a label in a given location, when Site 8 only sent one photo of a label attached to muffins. In all other cases we were sent multiple photos of shelf edges, menus, and tent cards. This was not a prespecified fidelity check criteria, nor was it ever planned as being a central indicator of implementation (proportion of total products with a label was the key indicator) but it was included to provide some more context to the implementation so readers could get an idea about the typical locations where the labels were implemented.

We have made this clear in the methods:

*“These photos were used to calculate the proportion of products that received PACE labels as well as the locations that the labels were presented. A site was coded as presenting PACE labels in a location (menus, shelf-edges, tent card, on products) if at least one photo was provided which showed evidence of a label in that location.”* P.12

**COVID-19. I'm a bit confused about the relevance of this section. An original plan was referred to, but this isn't in the pre-study protocol or in the clinical trials registry - in these study protocols visits were not planned. I do think a likely weakness of this study is that intervention fidelity could have been more accurately measured (i.e. study visits), hence why I think this section needs to be accurate.**

*Response:* We have removed this section as we agree that it is more confusing than it is helpful and we have extended the covid section of our limitations section to include this point.

*“These restrictions also meant that fidelity checks needed to be conducted via photographs taken by staff rather than site visits by members of the research team. The accuracy of the intervention fidelity measurements may have been affected by this, particularly if the photographs were did not include all products on sale at each site each day, which was not possible to determine without a researcher present.”* P.25 **Analyses
The analysis protocol specifies that total number of transactions would be controlled for in the primary analyses, but based on my reading of the manuscript this doesn't appear to be the case?
An aspect of the analysis I am struggling to understand is that 'to allow for linear trends, the day number of the trial was fitted as a continuous effect'  as was 'week of the trial'. Both day of the trial and week of the trial are going to be correlated with/confounded by intervention status, as days and weeks for intervention periods are on average going to be higher than non-intervention periods (i.e. I guess there should be a linear trend between trial day or week and absence vs. presence of the intervention), potentially resulting in multi-collinearity concerns. Is this the case?**

*Response:* Transactions were controlled for in the primary analyses, we have edited the text to make this clearer.

*“The number of transactions were included in the model as a proxy for site busyness.”* P.14

Time trends were modelled in a number of ways in the generalised additive model.

A) To allow for the different regular patterns due to day-of-the-week at each site, a random effect nested within site was included.

B) Further, to allow for any systematic shift week-by-week across all the data, a random term for the factor week number was included.

C) To allow for a linear time-trend during the study in a simplistic manner, a variable for the day number within a period was created and included as a fixed effect (e.g. for site 1 this was valued as 1-25 in the baseline period and 1-56 in the intervention period) – this could accommodate a potential underlying steady increase during the course of the whole study for example. This term was always non-significant.

In this way, we modelled known day-patterns in the data and allowed for other types of time patterns such as potential common linear time trends. In unreported analyses during model-building, the removal of any one of these time variables did not change the reported conclusions & results were stable. To clarify this in the text we have added:

**“**To allow for any potential linear time trend, the day number of the trial from the period start (e.g. for site 1 this was valued as 1-25 in the baseline period and 1-56 in the intervention period) was fitted as a continuous fixed effect. Model results were later found to be stable when these time variables were excluded one-by-one from the final model. The final model was chosen based on minimising the Akaike information criterion.” P.14

**In the full model reported in the supplementary materials (I would recommend the authors either summarise this in text in the main manuscript or provide it as a table) the effect 'Days from start on study period' presumably relates to days as a continuous variable from day 1 to week 1 to day 7 on week 12? (or does it relate to the first day of the intervention to the last day of the intervention for each site) and there is a significant negative trend - over the course of the 12 weeks, fewer kcals are ordered per day. I think this aspect of the data needs to be considered in more detail. The step design makes the implicit assumption that there is no reason effects should change over time until the intervention is implemented (i.e. mean intake before vs. during the step for each site is compared). However, there is a trend over time irrespective of the intervention (it appears). I appreciate the primary model controls for this, but as in my comment above I'm slightly
concerned about this approach because the change over time may well be being driven in part by the intervention always being introduced later in time. It would therefore be informative to analyse and report on whether this daily negative trend is a) observed across both the baseline and the PACE period to the same magnitude. If it is then my concerns above are probably not founded. If the trend differs between the two periods then this would be some evidence that the introduction of PACE labelling is changing kcal purchasing.**.

Response: First, we have added the full model from the supplement to the manuscript.

Second, we have clarified this time variable in the previous comment.

Third, the concern about change over time affecting an intervention introduced largely at the end of a study is one that is of course applicable to all stepped-wedge designs, but we have attempted to mitigate this in our modelling. The fact that the linear trend term was not significant is reassuring, so this point has already been addressed. We are happy to add this as a study limitation. Due to the pre-registered analysis plan, it would be inappropriate at this point to change to a different primary analysis being reported, hence we have kept the Bayes Factors as supporting evidence in the paper.

“A potential drawback of all stepped wedge designs is that time trends may be partially confounded with the intervention effect estimate however as linear time trend was no significant, this is less likely to be an important factor here.” P.24-5 **As they were planned and the study finds no effect from traditional significance testing, inclusion of the Bayesian analyses would be preferable.**

*Response:* We have now added this in.

**Page 17, line 1. I think cause and effect terminology should be removed as we don't know that the intervention significantly reduced energy purchased, we just know it's introduction was associated with a decrease.**

*Response:* We agree and have made this change.

*“Energy purchased was significantly lower during the PACE period in 4 cafeterias relative to baseline:”*

**Sensitivity analyses. These are well thought out and important to increase confidence in findings, so should be reported in the online supplementary materials for reviewers / readers to interpret.**

*Response:* As the key results from the sensitivity analyses are reported in the manuscript, we assume that the reviewer means the full models. We have added these to the supplement.

**Discussion
Page 18, line 25. It is the largest study on PACE labels, but this doesn't necessarily follow that it is the best estimate. There are limitations to this study and as the authors note there are likely to be contextual factors potentially moderating effects of labels (e.g. location etc.) which this study can't account for.  We also have no idea how well field studies translate from one country to another or one out of home food sector vs. another (e.g. Bleich et al. looked at PACE labels in convenience stores and therefore their study may be considered a better estimate because a lot of food purchasing happens in such stores), in the same way that it isn't clear how well lab studies translate from lab to real-world, so I would avoid terminology around this being the 'best' study.**

*Response:* While we would argue that our study better generalises to food and drink sales in general, as we applied labels to a range of food and drinks as opposed to just sugar sweetened beverages in Bleich’s study, we do acknowledge that it is not known whether our study results would generalise to different types of retail establishment and therefore we removed the second half of the sentence. The line now reads:

*“This is the largest study to date that evaluates the impact of PACE labels on food and drink purchases within a naturalistic setting* *with over 250,000 transactions collected via the electronic tills from 10 worksite cafeterias.”*

**Page 20, line 3. I'm not convinced that visual inspection does show that. Unless the authors can provide some exploratory analyses to support this point then I think this argument needs to be dropped. An alternative explanation is that effects are largest among studies with the largest imbalance between baseline testing period vs. intervention testing period? i.e. in studies in which one of the testing periods was only 4 weeks and therefore any difference between the two testing periods may be driven by shorter testing periods producing a less reliable estimate of what was happening in terms of consumer behaviour. This fits the data on individual site effects pretty much perfectly.  Is it a likely explanation? I don't really know and if the authors disagree then it highlights that making any inferences about why some sites appear to have larger effects than others based on just looking at what is similar/different between the sites is shaky ground.**

*Response:* We attempted to make our wording clear that this was speculative and hypothesis generating rather than a conclusion. However, to provide further evidence we conducted some exploratory correlations between 1) the effect of PACE on energy purchased from intervention items at each site and 2) the proportion (%) of kcals purchased from each food category at each site. See Table S2 copied below for convenience.

We have also amended the text to add this in:

*“Exploratory correlations suggest that PACE labels show larger effects in cafeterias that sell more discretionary items (e.g., savoury snacks, confectionery).”*

Table S2. Exploratory correlations between the effect of PACE on energy purchased from intervention items at each site and the proportion (%) of kcals purchased from each food category at each site (n = 10)

|  | Hot food | | Sandwiches | | Breakfast | | Bakery | | Confectionery | |
| --- | --- | --- | --- | --- | --- | --- | --- | --- | --- | --- |
|  | *r* | *p* | *r* | *p* | *r* | *p* | *r* | *p* | *r* | *p* |
| Effect of interventions | .63 | .053 | -.56 | .089 | .22 | .547 | -.11 | .759 | -.74 | .014 |

|  | Savoury Snacks | | Hot drinks | | Cold drinks | | Fruit and Veg | |
| --- | --- | --- | --- | --- | --- | --- | --- | --- |
|  | *r* | *p* | *r* | *p* | *r* | *p* | *r* | *p* |
| Effect of interventions | -.86 | .001 | -.72 | .019 | .06 | .863 | .43 | .763 |

*Note.* Negative correlations imply that the PACE interventions where more effective in cafeterias where they sold a lot of the products from the associated category.

**Page 21, line 3. Typo 'without'**

*Response:* Thanks for spotting this. We have corrected this:

*“Individual identifiers were not possible to acquire due to the companies’ desire not to share these data with an external organisation”* **Page 21. Line 18. I think there is better quality evidence to cite here which looks at change over time pre-post implementation of calorie labelling in restaurants (see Zlatevksa meta analysis for some of the studies) as opposed to comparing restaurants with vs. without kcal labelling cross-sectionally.**

*Response:* Thanks for pointing us to this analysis. We have adapted this section:

***“****However, a meta-analysis of calorie labelling studies suggests that retailers reduced the average energy content of their products by 15 calories after introducing the labels [20]. This suggests that PACE labels – which include calorie content – could have the same effect.”*

***** Reviewer #2:

My main comment relates to the absence of power calculation. Neither the protocol, SAP nor manuscript includes a power calculation. This raises questions about the a priori ability of this study to detect meaningful reductions in the primary outcome of interest.**

*Response:* We included a power calculation in the protocol, under the ‘Cafeterias’ subtitle (copied below for convenience).

*“We aim to recruit 10 sites, which would be more than similar calorie labelling studies in cafeterias (Vasiljevic et al., 2018, 2019). This would provide 80% power to detect a change in energy purchase of Cohen’s d = 1.00, using a before-after repeated measures design with 4-weeks in each period, using a 2-sided test and at the 5% significance level using a paired t-test.”*

But the reviewer is correct that we did not include this in the manuscript. Ultimately our recruitment strategy was based on practical limitations: the number of eligible sites that we could recruit, implement the intervention within, and collect sales data. We aimed to recruit more sites than this type of study typically does, which is around 4-6, but did not conduct a detailed sample size calculation. Instead our sample size calculation is a based on a simplified version of what we actually did, copied from the protocol. We thank the reviewer for noting this and have now amended the appropriate section of the manuscript to include this:

*“The recruitment strategy was based on practical limitations, specifically, the maximum number of eligible cafeterias that we could recruit from our collaborating company. We aimed to recruit 10 cafeterias, an increase compared to similar calorie labelling studies in cafeterias (Vasiljevic et al., 2018, 2019). An illustrative power calculation suggests this would provide 80% power to detect a change in energy purchase of Cohen’s d = 1.00, using a before-after repeated measures design with 4-weeks in each period, using a 2-sided test and at the 5% significance level using a paired t-test.”*

**Minor/specific questions:
-----------------------------

*       Please consider removing the results of the subgroup analysis by cafeteria from the abstract**

*Response:* This was the topic of lengthy discussions in our team prior to submitting the manuscript. The two main arguments essentially boiled down to against: the overall pooled estimate was our pre-registered primary outcome and adding in cafeteria-level effects distracts the reader from this, vs against: there was clear evidence from the cafeteria-level analyses (which were also pre-registered) that there was variation in effects, and that only providing the pooled estimate obscured important cafeteria-level variation.

Ultimately, we decided to report them and aimed to be clear in our language that the primary source of our inferences comes from the pooled result, but that there is also good reason to believe that exclusively focusing on this risks underplaying some potentially important aspects of these data.

We would prefer to keep them and we hope that our language is sufficiently clear to avoid a reader over-interpreting the cafeteria-level effects.

***       Please consider including p-values in the abstract.**

*Response:* We have added these in. ***       In the methods section, please include a power calculation including all the assumptions made.**

*Response:* We have now done this, as described above. ***       I note that the results of the primary analysis are reported as percentages when the outcome is total energy in kcal (absolute value). According to the analysis plan, available as a preprint, the outcome for the primary analysis is the absolute value (kcal), not a percentage change. If the intention is to take the baseline levels into account due to variations across cafeterias, I would suggest adding the baseline value as a covariate.  Please consider reporting absolute values instead or in addition to percentage changes. If presenting percentages, please clarify their meaning in the method/analysis section.**

*Response:* We have edited to the results to replace the percentage values with absolute values, although for the primary analysis we have left the percentage change in too for further context.

***       Please consider including a graph showing the absolute value of the primary outcome (y-axis) per week (x-axis) with one curve for each cafeteria. To indicate when each cafeteria switched to the intervention, one could use different colours or patterns. This would allow the reader to visualise the entire data series.**

*Response:* Thank you for this suggestion, we have created these and included them in the supplement, Figure S1*.*

***       Please note that analyses by individual cafeteria were unlikely to be sufficiently powered. When reporting the results (bottom of page 16 + top of page 17), please refrain from saying that the intervention significantly reduced or decreased xxx. It would better to write that "there was a significant difference/reduction/increase between the control and intervention period" without making a causal statement.**

*Response:* We agree with this suggestion and have amended the wording as follows:

*“There was no evidence that energy purchased was different during the PACE intervention relative to baseline in 5 cafeterias”* p.20

*“Energy purchased was significantly lower during the PACE period in 4 cafeterias relative to baseline”* p.20

***       Please add the outcome "total energy purchased" to table 3.**

*Response:* We have added this, and pasted the table here for convenience:

|  | Baseline | PACE |
| --- | --- | --- |
| Energy (kcal) purchased, per cafeteria from intervention categories | 148350 [87637] | 163949 [97267] |
| Total energy (kcal) purchased, per cafeteria | 149363 [113684] | 175858 [129821] |
| Number of transactions, per cafeteria | 315 [187] | 365 [204] |
| Revenue (£), per cafeteria | 979 [631] | 1166 [716] |

***       All outcomes are potentially affected by the number of persons with access to the cafeteria. I note that the analyses do not adjust for changes in exposure (number of persons) and wonder whether this has been / should be considered. It is potentially particularly relevant in the context of the Covid pandemic which may have impacted the number of persons "coming to work". In addition to potential fluctuations in the number of potential customers present, is it also possible that the profile of those present might have changed over time. Are sensitivity analyses possible?**

*Response:* We control for the number of transactions in the models which is defined as “the number of distinct payments to purchase products in the cafeteria, as a proxy measure for the number of customers per day” (see Covariates subsection of the method). We agree that controlling the number of people on site is crucial and the transactions variable gives us a good proxy measure of this as it includes all purchases made – including non-food and drink items such as newspapers, paracetamol, and tissues. Although it is impossible to know how many people walked into the cafeterias and did not buy anything, this transactions variable is the next best option for gauging on-site worker numbers. ***       The analysis model assumes a common time trend for all cafeterias. Given the apparent differences across cafeterias, it might make sense to run a model with varying time trends e.g. by adding a random slope by cafeteria (see Hemming, K., Taljaard, M. & Forbes, A. Analysis of cluster randomised stepped wedge trials with repeated cross-sectional samples. Trials 2017;18:101** [**https://doi.org/10.1186/s13063-017-1833-7**](https://doi.org/10.1186/s13063-017-1833-7) **). In addition, the effect of time was modelled as a linear time trend and I wonder whether this is a realistic assumption. One might wish to consider not assuming any trend e.g. by considering time/week as a class effect.**

*Response:* We thank the reviewer for this suggestion. As referenced in Hemming’s work, there are already random slopes by cafeteria included (as random slopes for week-day nested within cafeteria). Different time trend formulations were considered during modelling, and unfortunately, the extra parameters required caused models to drop numerical convergence and were not possible to include. We have added the following text to the methods to explain this point.

*“**Additional ways of modelling time trends were considered, but the above approach was adopted as more complex models stopped model fitting or led to boundary singularities”* p.14

**- Laurent Billot

*** Reviewer #3:

This study "Effect of physical activity calorie equivalent (PACE) labels on energy purchased in cafeterias: a stepped-wedge randomized controlled trial" was a stepped-wedge RCT of 10 worksite cafeterias in the UK to test the impact of PACE labels on total energy purchased from intervention labeled items per day compared to baseline. The study took place over 12 weeks between April 6 and June 28, 2021, which coincided with the time that the UK was implementing a phased re-opening of COVID-19-related restrictions. The study found that there was no overall effect of PACE labels on energy purchased but that there was variation of the effect between the different cafeterias.

This was a well-designed stepped wedge trial that had to be adapted somewhat due to the COVID-19 pandemic. The authors have done an excellent job of explaining the methods, the challenges to implementation, and the strategies to work around some of these challenges. The results are presented in a straightforward manner that is appropriate for demonstrating that there was no effect on the primary outcome.

My comments are therefore more directed at the introduction and discussion.  The introduction summarizes the existing literature on PACE labels quite thoroughly. The literature suggests that PACE labels may be effective for reducing energy intake, but there are relatively few studies of PACE labels conducted in real-world environments. However, I think the authors should be more explicit about why it was important for them to conduct their particular study.  What are the policy and/or program implications of using PACE labels in a worksite or other setting?  In other words, why is it important to do another study of PACE labels, and why in worksites? Despite multiple studies, PACE labels have not had much traction in the real world, and therefore it would be important to understand why the authors think this is an important research direction.**

*Response:* Thank you for your comments. To make our rationale clearer we have adapted the fourth paragraph within the introduction, p.5:

*“Based on the quantity and quality of evidence, considerable uncertainty remains about the effect of PACE labels to reduce energy purchased and consumed. Furthermore, there is evidence that effects reported in online or lab studies will be significantly smaller or even non-existent when implemented in naturalistic settings [7,12]. Even if an effect is replicated in a real world setting, the high variability in contexts make it hard to predict if an effect reported in one eating retail outlet will generalise to another.”*

This helps set up one of our existing points better in the fifth paragraph:

***“****The limitations of existing naturalistic studies are addressed in 3 ways: implementing the intervention in cafeteria settings across a wide range of food and drinks; collecting sales data from electronic tills to ensure the availability of data on every purchase made by every customer of the cafeterias throughout the study period; and, conducting the study in a larger number of sites to increase the study power and test the generalisability of the main effects to multiple cafeterias”* **I think the Discussion could include more discussion of the reasons why PACE labels may not be effective in the worksite setting. Much of the Discussion reviews food and cafeteria-level factors that may have influenced the effectiveness of the labels, but there is little discussion about employee-level factors that could have played a role.  For example, interpreting these labels requires both numeracy and health literacy skills. Even among highly educated populations, numeracy can vary greatly.  Other employee factors that might have influenced how the labels were interpreted or received include baseline physical activity level (e.g., employees with physically active jobs may not pay attention to these labels), weight/BMI, race/ethnicity, SES status, and gender. Finally, the impact of the pandemic on employees' attention and motivation to making healthier food choices could have contributed to a lack of effectiveness; while this was suggested in the limitations, I think
this the pandemic was a major factor during the study and could be elaborated in more detail.**

*Response:* We agree with the reviewer that these are a number of possible explanations for the null results and we have amended out discussion slightly to make this clearer. We were cautious about developing this point too much however, as these are just hypotheses at this stage and we didn’t want to overstate the likelihood that one of these factors was the reason behind the null results:

*“There may also have been individual differences in those using the different cafeterias, which modified their responses to PACE labels. These differences include demographic characteristics including age, gender ethnicity, socio-economic status, or other factors such as numeracy (for interpreting the values) or weight status.” P.23*

*“Although this was not tested, this could include the pandemic affecting food selection or a preference for takeaway foods from cafeterias to avoid crowded spaces.”* P.25

**Finally, a limitation of PACE labels (and calorie labels in general) is that they only focus on energy content, rather than the dietary quality of the food; therefore, people who are not focused on weight loss or on preventing weight gain may not think that PACE labels are relevant to them.**

*Response:* We have added this point into the Discussion:

*“A further concern is that PACE labels focus on energy content, rather than other characteristics of the food, they may be ignored by people who are not attempting to lose or maintain their weight.”* P.26

**The limitations should include a comment about the generalizability of the results for other worksite or retail settings that may serve more socioeconomically and racially/ethnically diverse populations.**

*Response****:*** Although we couldn’t get data on the proportions of employees by socio-economic position, the sites included workers with a wide range of occupations including pilots, accountants, managers, drivers, cleaners, administrators, sales, and manufacturing staff (see Table 1). In addition, we don’t have any data on ethnicity. We have edited the Discussion to cover these points:

*“**Contextual factors may have modified the impact of the labels, such as the type of food and drink sold at the cafeterias, the degree of label implementation, or whether the cafeteria is mainly used to eat-in or take away.* *There may also have been individual differences in those using the different cafeterias, differences which modified their responses to PACE labels. These differences include demographic characteristics including age, gender ethnicity, socio-economic status, or other factors such as numeracy (for interpreting the values) or weight status.”*

***** Reviewer #4:

The manuscript assessed the effect of PACE labels on purchased on 10 food outlets. The strength of the study is the natural setting in which the experiment was conducted. The manuscript is clearly written. There are important methodological limitations that may affect the quality of the study and challenge the validity of the findings.

An important limitation, that has remained unaddressed, is the power of the study. With a clustered design, the power of the study to detect the anticipated effect remains unwarranted. It is quite conceivable that the study was underpowered to assess the (possibly small) effects of the labels.**

*Response:* We agree with this point. In response to Reviewer #3, we have added the *a priori* sample size calculation from the protocol. Ultimately, as there was limited information on exactly this type of intervention within this specific context sample size planning was mainly based on pragmatic considerations of resourcing, which we have made clear in the added sample size statement and does not require also adding to the limitations.

*“The recruitment strategy was based on practical limitations, specifically, the maximum number of eligible cafeterias that we could recruit from our collaborating company. We aimed to recruit 10 cafeterias, an increase compared to similar calorie labelling studies in cafeterias (Vasiljevic et al., 2018, 2019). An illustrative power calculation suggests this would provide 80% power to detect a change in energy purchase of Cohen’s d = 1.00, using a before-after repeated measures design with 4-weeks in each period, using a 2-sided test and at the 5% significance level using a paired t-test.”*

**A related concern here, is the potentially different effect that the label has on different food groups. It is unfortunate that the study did not assess effectiveness of the label by different categories of food/beverage items. The authors provide evidence in the manuscript that the labels have previously shown effects on specific categories of food items (e.g., sugar sweetened beverages). The hypothesis that the introduction of the labels would influence purchases of a wider range of food items was possibly too optimistic. Further detail on how the labels have led to changes by categories of food items and/or introduced a shift in purchases between different categories would have been an informative to understand the potential effectiveness of PACE labels on food and beverage purchases.**

*Responses:* The main reason for testing the effect on as many foods and drinks as possible was to extend the limited data from sugar sweetened beverages, to see if the effects are detected across a broad range of products. We would argue that the most important question is whether the labels work on aggregate as if the labels are ever implemented, it is likely that they would be added to any food and drink product where the energy content is known, which is what we did here. We do agree with the reviewer however, that it is a very important secondary question to investigate whether the labels are more or less effective at changing the purchasing of specific products. We speculate on this in the Discussion based on the data that we have, and we are conducting a follow-up study to investigate this further.

*“Table S1 in the supplement shows that the cafeterias varied largely in terms of the food and drink that were regularly sold. For example, the proportion of energy purchased from hot meals ranged from 2% to 27%, breakfasts from 5% to 58%, sandwiches from 4% to 19%, and hot drinks from 0% to 13**%. Exploratory correlations suggest that PACE labels show larger effects in cafeterias that sell more discretionary items (e.g., savoury snacks, confectionery). It is possible that PACE labels are more effective at reducing purchasing of discretionary items compared to main meals, which may explain why the effect sizes are larger at certain sites. Further research is needed to confirm this.”* **An additional limitation to the study is information on whether the customers have seen and understood the labels. Some exist surveys or assessment of the use of labels in the shops would provide important additional information to interpret the findings.  It remains unclear how the label was developed and tested in the population. A formative study on how the target population understands and interprets the labels would provide the necessary evidence to support the proposed hypothesis tested. Without this information, it is quite possible to assume that the customers simply did not see or understand the labels during their decision-making process in the shops.**

*Response:* We developed the labels based on existing templates that have been studied extensively in online studies. We agree with the reviewer that their suggestion would be essential if this was a new type of label. But this was not the case. We used a label that has been administered to over 5000 participants in previous studies (from the Daley meta-analysis: <https://jech.bmj.com/content/74/3/269.abstract?casa_token=J-R3keJLcGMAAAAA:61bYxgSCAegcqRPQG6QoR8UXbqaPS7hJw9bSmh5tuAJp07PEityLcDRGS2oamUijSt_K4Kcj6_A>). To develop the labels we took several designs from previous research which uses different variations of activity (e.g., running, swimming, walking) and quantity (minutes, miles) however, previous research has not found that one is better than another, so we selected the one we judged to be most accessible and easily understood: walking and minutes. We have added this information into the intervention section in the method:

*“Previous studies have evaluated different variants, for example running vs walking, and minutes vs miles. In the absence of any evidence of their relative effectiveness, the investigators selected the design they judged to be the most accessible and easily understood, namely communicating the number of minutes the average person would need to spend walking to expend the energy contained in the product.”* P.9

Once we had these initial versions we sent them to the catering staff and managers at the different cafeterias who provided feedback over email and in video-call meetings. We went through several iterations of designs until we settled on the one that we used. We have expanded this section to make this process clearer:

*“Initial versions were developed by the research team based on designs reported in previous published papers before getting feedback from the catering staff and managers working at the study sites. After several iterations we agreed on the selected design.”* P.9

**The CONSORT checklist is rather of limited use (e.g. no pages numbers are provided) and lacks detail. E.g., authors simply refer to page "Cafeterias, Para 2" for a description of sample size. No critical considerations on power and sample size are found there.**

*Response:* The editors of Plos Medicine recommend this format as page numbers are not found in the final version. **A description of the food offer in the different outlets would be helpful, including a description of other, existing, labels that are used on the food and beverage items.**

*Response:* Apologies that this was not clearer, this information is available in the supplement, Table S1. We have added a new sentence into the method to guide readers to this information:

*“For further information on the products sold at each site, see Table S1 in the supplement.”*
