# Supplementary material for: Evaluation of physical activity calorie equivalent (PACE) labels’ impact on energy purchased in cafeterias: A stepped-wedge randomised controlled trial
Source: PLoS Med. 2022 Nov 8;19(11):e1004116. doi: 10.1371/journal.pmed.1004116 (PMC9642872; doi:10.1371/journal.pmed.1004116)
Supplement: Attachment — Submitted filename: Responses to reviewers 17.09.22.docx [file pmed.1004116.s005.docx]

**Editor comments**

We thank the reviewers and editors for their comments, addressed below. We also noticed another item that needed changing following the previous resubmission where the effects in percentage terms were changed to kcals per transactions. We selected the kcals per transaction originally as we felt it was more interpretable, however it is not consistent with our preregistration which states kcals change not kcals per transactions change, so we have added in the effect per site, as per the preregistration, and then added in “equivalent to X kcals per transaction…” to provide further details to the reader to aid interpretation. We also spotted a slight error in the original *per transaction* calculations, so the values are slightly different (eg the main effect goes from -7 kcals per transaction to -5 kcals per transaction, however the p values and conclusions are all unchanged and unaffected by this error). For example, an extract from the abstract:

“Regression models showed no evidence of an overall effect on energy purchased from intervention items, -1934 kcals per site per day (95% CI -5131 to 1262), *p* = .236, during the intervention relative to baseline, equivalent to -5 kcals per transaction [95% CI -14 to 4].”

**Please adopt a cautious approach to the by-site observations, as requested by the referees.**

*Response:* There were two mentions of the by-site observations below, one from Reviewer #2 about including the results in Table 3 which we have responded to below and a second from Reviewer #3 about removing them from the abstract which we agreed with and have removed.

**Early in the abstract, we ask you to adapt the text for greater clarity. The second sentence could be adapted to begin "However, the meta-analysis included only 1 study conducted ..."; with the subsequent sentence becoming "We therefore aimed to estimate ... in the context of a randomized study design.", or similar.**

*Response:* We have made these changes. The new section is copied below:

*“A recent meta-analysis suggested that using physical activity calorie equivalent (PACE) labels results in people selecting and consuming less energy. However, the meta-analysis included only 1 study in a naturalistic setting, in 4 convenience stores. We therefore aimed to estimate the effect of PACE labels on energy purchased in worksite cafeterias in the context of a randomised study design.”*

**Again at line 10, we suggest "... was carried out to investigate the effect of PACE labels ...".**

*Response:* We have made this change.

**Immediately after the abstract, please include a new and accessible 'Author summary' section in non-identical prose. You may find it helpful to consult one or two recent research papers published in PLOS Medicine to get a sense of the preferred style: we generally expect three subsections, each of around three bulleted points, each point consisting of one or two short sentences (usually not repeating quantitative elements already quoted in the abstract).**

*Response:* We have completed an Author Summary section and provided this below:

**Author Summary**

**Why was this study done?**

- Overconsumption of energy from food and drink contributes to the high and rising prevalence of obesity
- PACE (Physical Activity Calorie Equivalent) labels have been suggested as one intervention that could lower consumption of high energy foods

**What did the researchers do and find?**

- We carried out a randomised stepped-wedge trial over 12 weeks
- PACE labels were added to a range of food and drinks in ten cafeterias after a baseline period
- The labels did not significantly change overall energy purchased in the cafeterias, although there was considerable variation in the effects between cafeterias

**What do these findings mean?**

- There was no evidence of an overall effect of PACE labels on energy purchased in cafeterias in this sample
- There may be important contextual factors that influence when PACE labels reduce energy purchased

**If not done already, please refer to the attached study protocol in the Methods section (main text).**
*Response:* We have added this in:

*“The study protocol and CONSORT extension checklist for stepped-wedge trials are attached as supplements (see “S1_CONSORT_Checklist” and “S2_Study_Protocol”)”*

**Please rename figure 1 "Participant cafeteria inclusion flowchart" or similar.**

*Response:* We have renamed this: “Cafeteria inclusion flowchart”

**In the Results section (main text), under "Total energy purchased (intervention and non-intervention items)" we ask you to revisit the phrase "... indicating that it is more likely that this intervention decreased ...". To avoid the issue of "more likely than what?", we suggest rewording to "... suggesting that the intervention may have led to a decrease in total energy purchased ...".**

*Response:* We believe the suggested line could lead the reader to a conclusion that isn’t fully supported by these data. We have therefore removed this additional line altogether:

*“There was no clear evidence that PACE labels changed the total energy purchased from all products, -10 kcals per transaction [99.5% CI -20 to 0],* *p = .051.~~, albeit with the upper CI estimate touching zero indicating that it is more likely that this intervention decreased total energy purchased from all products.~~ “*

**We suggest beginning the Discussion section (main text) with "In this study, we found ..." or similar.**

*Response:* We have made this change:

***“****In this study, we found no overall evidence that PACE labels changed energy purchased…”*

**In the subsequent paragraph, please avoid or qualify "This is the largest study to date".**

*Response:* We have added a qualification:

*“This is the largest study to date – to our knowledge – that has evaluated the impact of PACE labels...”*

**For reference 1 and any other relevant citations, we suggest spelling out "GBD".**

*Response:* We have made this change

**Noting reference 2, please list no more than 6 author names in each citation throughout the reference list, followed where appropriate by "et al.".**

*Response:* We have made this change

**Can a publisher and URL (with accessed date) be added to reference 6?**

*Response:* We have made this change

**Immediately after the reference list, "S1 PRISMA checklist" should surely refer to CONSORT.**

*Response:* We have corrected this error:

**“S1** CONSORT Checklist. Checklist of information to include when reporting a stepped wedge cluster randomised trial (SW-CRT) (PDF)”

**Thank you very much for including the completed CONSORT checklist. We note that the entries include "Random", for example, and in the interests of clarity we ask you to expand on these entries (e.g., "Methods: section on randomization"). Converting this to a Word doc may help.**

*Response:* The entry “random” was cut off, as the pdf text box only displays a portion of its contents and you have to scroll through to see the whole content. However, we agree that this isn’t user friendly so have converted the file to a Word document so the contents are more accessible.

**Comments from Reviewers:

*** Reviewer #1:

The authors have responded to my comments comprehensively, aside from one point.

I am still very unconvinced about their speculative suggestions regarding why some outlets may show an effect and others don't. The exploratory analysis is a series of 9 additional correlations without any adjustment for multiple comparisons using a tiny sample size (n=10). The problem with very small sample sizes is that they are only powered to detect very large and infeasible effect sizes. Munafo et al. have written about this. Others have too -** [**https://onlinelibrary.wiley.com/doi/10.1111/brv.12315**](https://onlinelibrary.wiley.com/doi/10.1111/brv.12315) **A very conservative adjustment for the large number of analyses conducted (p = 0.01) would render one of the significant effects non-significant and the other significant effect is for an r = -0.86. It is inconceivable that the true size of relationship would be anywhere near that large. As I noted, there would probably be a negative correlation between imbalance between baseline testing period vs. intervention testing period and site effect size. But because it may well be spurious and is just post-hoc guess work based on data / observation fishing from a tiny sample size, I wouldn't ask the authors to run a correlation as I wouldn't have any confidence in the result. Given how tiny the sample size is for these analyses (and therefore how unreliable any analysis would) and how infeasible the result is, I don't think it is questionable/misleading to draw any meaning from it.**

*Response:* We completely agree with these points about the correlations. We included them only to provide some statistical support to our response in the last round of revisions about visually inspecting the tables which was challenged. Our goal here was never to reach a conclusion from the correlations or from visually inspecting the tables, but rather for it to be a hypothesis generating approach so that future research could confirm or refute this. We have reworded this to make it clearer: p.23

*“Exploratory correlations suggest that PACE labels show larger effects in cafeterias that sell more discretionary items (e.g., savoury snacks, confectionery). It is possible that PACE labels are more effective at reducing purchasing of discretionary items compared to main meals, which may explain why the effect sizes are larger at certain sites. Although the sample size is too small for any conclusion to be reached from these correlations, it does suggest a testable prediction for future research.”*

***** Reviewer #2:

My comments have been adequately adressed. I am not convinced about including the site results, especially the "modelling of the variance" part, in Table 3 but will leave the decision to keep them with the editors.
-Laurent Billot**

*Response:* Thank you for this comment. Using generalized additive models allows parameters for the variance (or higher moment terms), allowing sites with different levels of variability to be appropriately included in one model. For example sites of appreciably different sizes. The “modelling of the variance” terms in Table 3 are significantly negative, meaning there was significantly less variability compared to Site 1. Since the model diagnostics were reasonable, we argue this is clearly an advantageous way to model the data which accounts for sources of variability which are left as unknown in other types of modelling.

*** Reviewer #3:

**I commend the authors for being highly responsive to reviewers' comments in their response document and in the manuscript edits.

I have just a few additional comments/concerns about the Abstract:
1) The methods and findings should clarify that the primary and secondary outcomes are changes from baseline in total energy (kcal) purchased from intervention items, non-intervention items, and overall purchases per day. "Change from baseline" is not currently specified in the abstract.**

*Response:* We have edited this to add “… relative to baseline” into the abstract.

**2) The findings of increased revenue per transaction is statistically significant, but the relevance to policy or intervention implementation is not discussed in the abstract, or for that matter, in the manuscript.  At the very least, I can't see a good reason for highlighting this finding in the abstract because it does not seem meaningful, and the authors do not include any discussion about this finding in the discussion section of the manuscript. I think it should be removed from the abstract.**

*Response:* We have removed this from the abstract.

**3) I would also consider removing from the abstract the references to the site-specific findings, including the sentence, "Of the 10 cafeterias, there were null results in 5, significant reductions in 4, and a significant reduction in 1" and the conclusion that "There was considerable variation in effects between cafeterias, suggesting potentially important unmeasured moderators." Highlighting the findings of the individual cafeterias seems to counter the authors' main justification for conducting their multi-site PACE study that is described in the Introduction of the manuscript. In the last paragraph of the Introduction, they state, "The limitations of existing naturalistic studies are addressed in 3 ways. . . [including] conducting the study in a larger number of sites to increase the study power and test the generalisability of the main effects to multiple cafeterias," and in the second to last paragraph of the Introduction, they suggest, "Even if an effect is replicated in a real world setting, the high variability in contexts make it hard to predict if an effect reported in one eating retail outlet will generalise to another." Given these strong arguments for the need to include multiple sites to be able to determine if PACE labels are effective and generalizable, I do not think the findings for the individual worksite cafeterias should be featured in the study abstract.**

*Response:* Following some discussion we have decided to remove the site-level results from the abstract.

***** Reviewer #4:

Thank you for addressing my comments and updating the document.

*****

Thank you for your comment.
